# Elasticity and Dynamics of Elastomeric Epoxy Networks: Comparing Simulations and Experiments at High Frequency

**DOI:** 10.1021/acsmacrolett.5c00517

**Published:** 2025-11-08

**Authors:** Iakovos Delasoudas, Spyros V. Kallivokas, Emmanouela Filippidi

**Affiliations:** † Dept. of Mechanical Engineering and Aeronautics, 123277 University of Patras, Patras, 26500, Greece; ‡ Computation-Based Science and Technology Research Center, 338376 The Cyprus Institute, Nicosia, 2121, Cyprus; § Dept. of Materials Science and Engineering, 37777 University of Crete, Heraklion, 70013, Greece; ∥ Institute of Electronic Structure and Laser, FORTH, Heraklion, 70013, Greece

## Abstract

This study investigates the elastic and dynamic properties of elastomeric, stoichiometric epoxy networks formed between the telechelic functionalized poly­(ethylene glycol) diglycidyl ether (PEGDE) and the linear cross-linker 1,4-diaminobutane across a range of extensional strain rates (10^7^ to 10^10^ s^–1^), molar masses (*n* = 3, 5, 8 repeat units), and two reaction extents determining degree of cross-linking through atomistic simulations and compares them with the experimental *n* = 8 system. Investigated properties are Young’s and shear moduli, the *C*_11_ elastic constant, the glass transition temperature, and the network’s mean-squared-displacement. Results reveal a notable agreement between simulation-obtained and experimental values of *C*_11_ and its experimentally determined Brillouin light scattering (BLS) value and glass transition temperatures, bridging the gap between atomistic and macroscopic length scales. This work contributes to the renewed interest of BLS applied on soft systems and lays the groundwork for computational investigations of complex epoxy architectures, such as dual networks with epoxy covalent and noncovalent bonds.

## Graphical abstract

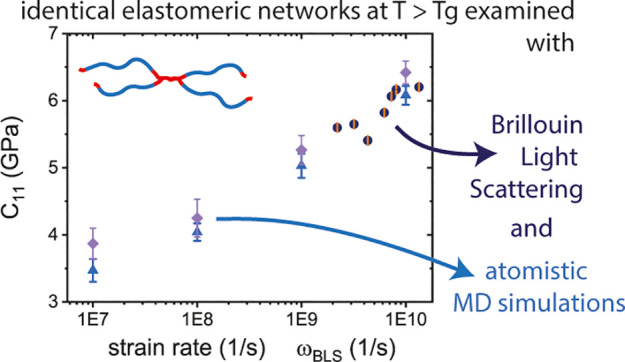

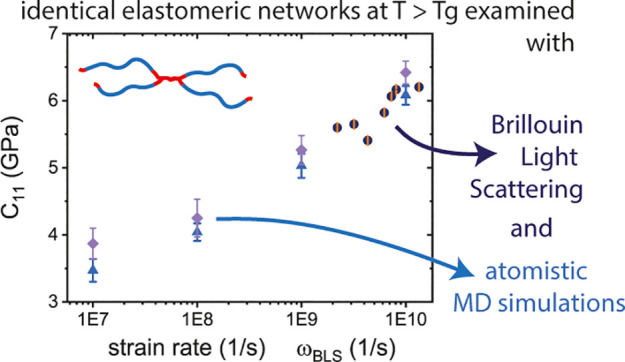

Molecular dynamics (MD) simulations are widely used to study the elastic, thermal and fracture^1–6^ properties of dry epoxy and epoxy-based networks,^7–9^ which are ubiquitous in industrial applications due to their consistent production of tough, chemically resistant, colorless thermosets. Although MD offers unparalleled molecular-level insight and enables access to time and length scales not readily probed by experiments, a significant limitation remains in its accessibility to strain rates in comparison to material testing. Uniaxial tensile simulations typically operate at strain rates on the order of 10^7^ s^–1^, which far exceed the 1–10 kHz frequency range characteristic of experimental techniques such as rheology or dynamic mechanical analysis (DMA). This leads to extrapolations and comparisons of mechanical properties measured at least 3 to 6 orders of magnitude apart.^2,5,10^ In this work, motivated by the renewed interest in Brillouin light scattering (BLS) applied to soft and biological materials^11–14^ as a way to probe material mechanical properties, and efforts in other materials science fields to combine novel computational techniques with BLS,^15^ we set out to compare the MD results of mechanical, uniaxial testing simulations on a soft elastomeric system against experimental data from BLS that lie in the same order of magnitude probing frequencies. While the underlying physics of the two approaches are different, as the former measures directly mechanical properties, while the latter measures the frequency of acoustic phonons and, in particular, the frequency shift of the scattered light, which is subsequently transformed into stiffness metrics, the frequency range in which they both apply is identical. Thus, we posed the question whether simulations and experiments may provide matching results at high frequencies, as opposed to comparisons across a 6 orders of magnitude frequency gap.

In contrast to industrial epoxy thermosetting resins that exhibit glass transition temperature (*T*_g_) above 100 °C, rendering them glassy at room temperature, our system of choice is a soft, elastomeric epoxy network with *T*_g_ significantly lower than room temperature. This is of practical interest, avoiding the need to resort to long, viscous, and hard-to-process polymers and is closer to model systems for nonhydrogel biological materials.

Herein, we study dry networks of linear poly­(ethylene glycol) diglycidyl ether (PEGDE) of *M*_n_ = 500 and 1000 g/mol with the linear tetrafunctional cross-linker 1,4-diaminobutane (DAB or putrescine, Figure 1) that exhibit *T*_g_ < *T*_room_,^16,17^ resulting in elastomeric epoxy networks. Although tetrafunctional diamine cross-linkers such as diethyl toluene diamine (DETDA, Figure 1) are industrially preferred and DAB has been avoided due to its odor prior to cross-linking and high reactivity during, it has recently gained renewed attention due to the achievement of biobased production with efforts for industrial-scale bioproduction.^18,19^ PEG is biocompatible and hydrophilic, and as such various PEG-based hydrogels^20^ have also been experimentally explored for biomedical uses.

**Figure 1:**
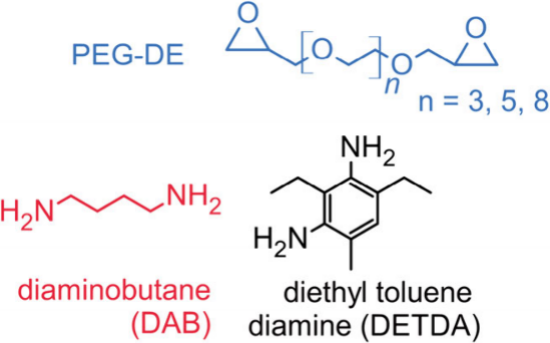
Molecular structures of the PEGDE and linear DAB used in this study and DETDA, one of the typical industrial cross-linkers.

Stoichiometric PEGDE/DAB cross-linked dry networks of PEGDE 500 and 1000 g/mol precursors, respectively named PEG-X500 and PEG-X1k, were found to have^16^ reaction extents over 98%, respective *T*_g_ values of −40.6 °C and −50.2 °C by DSC at 10 K/min, and largely frequency and temperature independent *G*′ ∼ 1.78 and 0.8 MPa at low frequencies (10^–1^ to 10^2^ rad/s) via oscillatory rheology. Conventional rheology was complemented^14^ with piezo oscillatory rheology up to 7·10^3^ rad/s and by BLS in the GHz range. Thus, experimental data from molar mass identical with those of the MD simulations will be compared.

Experimental network synthesis of the relevant PEG-X500 (*n* = 8–9) and PEG-X1k (*n* = 20) networks has been described elsewhere.^14,16^ For the fully atomistic MD simulations, three distinct networks were created from the bifunctional PEGDE of *n* = 3, 5, and 8 mixed with the tetrafunctional DAB (Figure 1) in a functional group stoichiometric ratio, leading to a 2:1 PEGDE:DAB molar ratio. The molecular ratio, 128:64, was kept fixed throughout. The well parametrized interatomic potential PCFF-IFF^21^ was chosen, as it has previously proven to accurately predict physical, mechanical, and thermal properties of polymers.^22–24^ All the configurations, file preparations and force field application on the systems, pre and post cross-linking reaction were constructed with the open-source Python code LUNAR.^25^ For each system of *n* = 3, 5 and 8, three statistically independent configurations were generated.

Initially, all PEGDE and DAB molecules were packed in a low-density simulation box (0.2 g/cm^3^). Subsequently, energy minimization was performed using the conjugate-gradient method in the Large-Scale Atomic/Molecular Massively Parallel Simulator (LAMMPS)^26^ with an energy tolerance of (10^–6^) (unitless), a force tolerance of 10^–6^ kcal/mol/Å, a maximum of 500 iterations, and a maximum of 10,000 force/energy evaluations. Each replicate was equilibrated in the constant pressure and temperature (NPT) ensemble at temperature 27 °C and pressure 1 atm with a time step of 1 fs for a total of 500 ps. The Nosé-Hoover thermostat and barostat^27^ were employed for all MD simulations. To evolve the low-density systems to their target bulk mass density, the “fix/deform” command in LAMMPS was used to gradually reduce the simulation box volume at 27 °C over 5 ns, reaching a final density of 1.20 g/cm^3^). After densification, an annealing cycle further relaxed the configurations by heating to 227 °C, followed by cooling back to 27 °C at a rate of 20 °C/ns in the constant volume temperature (NVT) ensemble, followed by a 1.5 ns equilibration in the NPT ensemble at 27 °C and 1 atm prior to cross-linking. All simulations used a 1 fs time step. The final densities for all systems were around 1.11 g/cm^3^, approaching the experimental density of 1.1 g/cm^3^ of PEG-X1k and 1.15 g/cm^3^ of PEG-X500.

Epoxide-amine cross-linking was implemented by an in-house code based on the work of Li and Strachan^28^ combined with the REACTER protocol^29,30^ “fix bond/react” in the open-source code of LAMMPS. Cross-linking was simulated at 227 °C in the NVT ensemble with a time step of 0.5 fs using the REACTER protocol^30^ which in the two-step reaction involves the creation of two pre- and two post-reaction templates with their corresponding mapping files. For bond formation to occur, the minimum cutoff distance was set to 6 Å and the bond formation probability at 0.5. During cross-linking, every 20 ps a minimization step and a velocity reset with conserved linear and angular momentum prevented the system from drifting out of the simulation box. For *n* = 3, 5, 8, cross-linking terminated when the amine reaction extent (RxE) reached ∼ 82%. To better address comparisons with the experimental PEG-X500, an additional system of *n* = 8 and RxE = 92.5% was created, where all molecules were cross-linked in a sole percolating cluster. Network measures are shown in Tables 1 and S1, where primary amines denote the unreacted ones, secondary those after one reaction, and tertiary after both reactions. Notably, the *n* = 5 networks contain slightly more secondary amines, implying a higher content of elongated chains. Such observed heterogeneity in the simulated system is on par with heterogeneity in the experimental system. After cross-linking and before initiation of mechanical characterization, all systems were cooled down to 27 °C and equilibrated for 100 ns with a 1 fs time step at 1 atm under the NPT ensemble.

**Table 1:** Characteristics of Crosslinked Networks

		Amine characterization		
PEG-DE, *n*	RxE^a^ (%)	Primary	Secondary	Tertiary
3	82.4	17 (13.3%)	16 (12.5%)	95 (74.2%)
5	82.7	11 (8.6%)	24 (18.8%)	93 (72.7%)
8	82.4	14 (10.9%)	17 (13.3%)	97 (75.8%)
8	92.5	4 (3.1%)	11 (8.6%)	113 (88.3%)

a RxE = reaction extent.

For the mechanical characterization, systems were subjected to uniaxial tensile simulations across all three directions (*x*, *y*, *z*) and shear deformation simulations in the *xy*, *xz*, and *yz* planes and subsequently averaged,^3,4,23^ under four different strain rates: 10^7^, 10^8^, 10^9^, and 10^10^ s^–1^. Uniaxial tensile tests were conducted under the NPT ensemble, allowing the manifestation of the Poisson effect. Young’s modulus, *E*, was calculated (Figures 2A, S1, Table S2) from the stress–strain curve for elastic strains up to 3%. Decreased stiffness for higher molar mass precursor PEGDE is observed for all strain rates, as expected due to decreased cross-link density.^2^ The linear strain rate dependence of *E* vs log­(strain rate) is notable in Figure 2A. Whereas experimentally the expectation is for *E* to reach a plateau at such high strain rates, in MD simulations this should only be expected at rates 10^12^–10^14^ s^–1^ that are comparable to atomic bond vibrations. This is a computational feature of MD simulations: in order to be strain rate independent, deformations must be applied with ultrahigh strain rates that surpass the atomic thermal vibrations.

**Figure 2:**
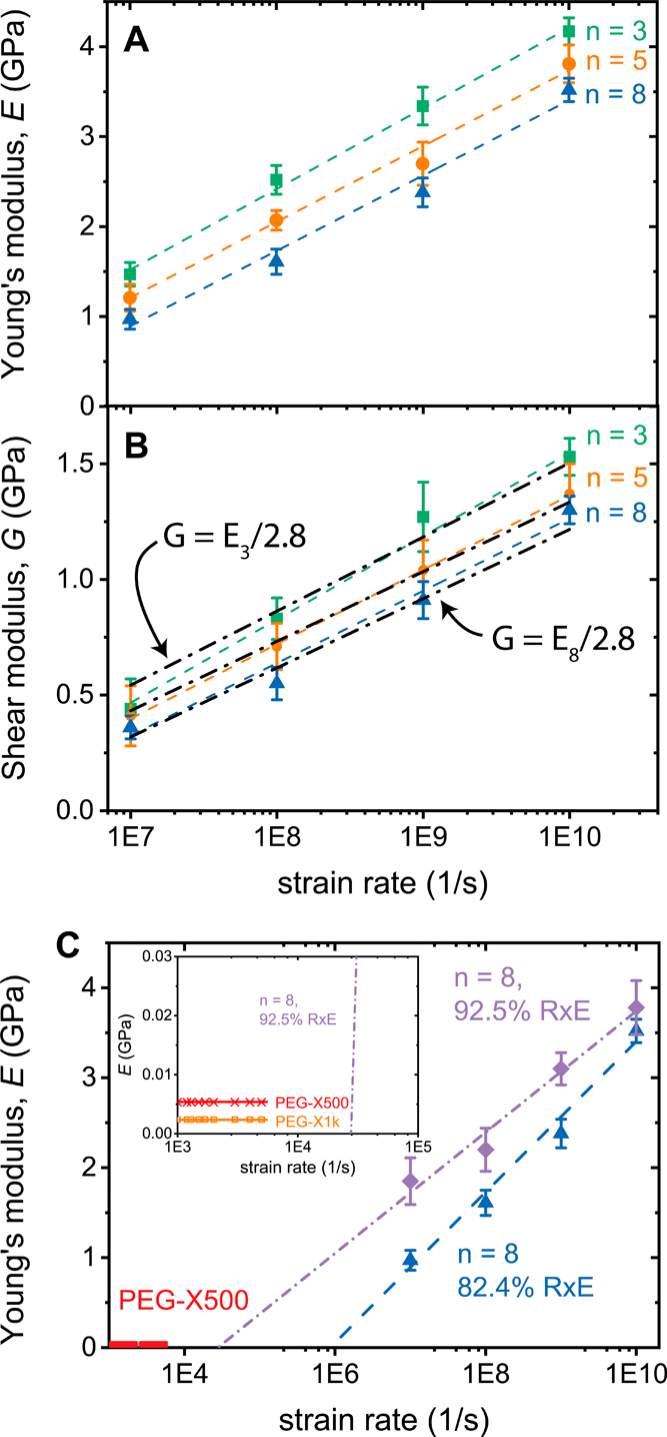
Network molar mass and strain rate dependence of (A) Young’s modulus, *E*, and (B) shear modulus, *G*, for *n* = 3 (green squares), *n* = 5 (orange circles), and *n* = 8 (blue triangles). Colored dashed lines are linear fits to the data. In (B), black dashed-dotted lines are the prediction of *G* = *E*/2.8 (ν = 0.4) instead of *E*/3 8 (ν = 0.5) using for *E* the fitted lines from (A). (C) Comparison of Young’s moduli between the *n* = 8 networks with different degree of cross-linking. Linear fits and extrapolation were performed until *E* = 0. In red, calculated *E* values from rheology for the experimental systems PEG-X500 and (inset, orange) PEG-X1k.

In Figure 2B, the directly measured shear modulus, *G*, is shown, overlaid with the calculated *G* (black dash-dot curves) from the *E* fits of Figure 2A, using equation

G=E/2(1+ν)=E/2.8

with Poisson’s ratio ν = 0.4, which better matches the curves than ν = 0.5. The directly MD-calculated and averaged Poisson’s ratios ν_*ji*_ = – *ε*_*i*_/*ε*_*j*_ are 0.41 ± 0.08 for *n* = 3, 0.42 ± 0.11 for *n* = 5, and 0.36 ± 0.09 for *n* = 8 at strain rates 10^8^ s^–1^ and practically identical for 10^7^ s^–1^, and within one standard deviation are close to ν = 0.5. While typically ν = 0.5 and *G* = *E*/3 are expected for elastomers, it is not untypical for MD literature-reported ν values to fall short for epoxy resins in the glassy regime^3,31^ In MD simulations, one source of error is the larger standard deviation values for *G* than *E*, attributed to (i) the involvement of off-diagonal stress components which tend to be noisier than normal stresses used in *E*, (ii) smaller absolute values, and (iii) smaller than ideal MD cells which exaggerate fluctuations resulting in larger direction-dependent shear components. If we compared the MD-measured *n* = 8, RxE = 92.5% *G* = 1.9 GPa at 10^7^ s^–1^ with the shear modulus from rheology, *G*′ ∼ 1.78 MPa,^14^ measured at 3–4 orders lower frequency, the discrepancy, as expected, is vast. Extrapolating the linear relationship *E* vs log­(strain rate) (Figure 2C, purple), crosses *E* = 0 at 2.7 × 10^4^ s^–1^, which although possibly acceptable for predicting the upturn of the low-frequency regime around 10^4^ s^–1^, fails to capture the plateau and largely frequency-independent values persisting for four/five decades down to 10^–1^ s^–1^.

The main result of this study is the direct comparison with experimental measurements at high frequencies.^14^ As such, the stiffness matrix elastic constants *C*_*ii*_ were directly calculated by uniaxial tensile simulations, keeping the lateral dimensions of the box fixed. For isotropic systems, *C*_11_ = *C*_22_ = *C*_33_, and thus the reported *C*_11_ is the averaged value of the three *C*_*ii*_. Experimentally, for a homogeneous, isotropic, linear elastic solid under small strain, the longitudinal modulus of a Brillouin scattering experiment is the measurement of the stiffness from the longitudinal acoustic mode, which is *C*_11_: *M*′ ≈ *C*_11_. In previous work,^14^ *M*′ was measured via BLS for the experimental systems PEG-X500 and PEG-X1k, as shown in Figure 5B therein.

Although there is no formal equivalence between mechanical, uniaxial, high strain rates and oscillatory, GHz-frequency of BLS acoustic phonon propagation, we propose a mapping between them as an ansatz based on their shared underlying physics: both probe linear response and segmental dynamics (as opposed to full chain dynamics). While such parallelism is established in the glassy regime *T* < *T*_*g*_, we are herein attempting the extension to elastomeric systems at *T* > *T*_*g*_ on the grounds that such high frequencies are sufficiently high for glassy effects to manifest. The dispersion of *C*_11_ is also due to segmental relaxations. Furthermore, in measuring *E* at such high strain rates, chains are not given sufficient time to relax, so again, the instantaneous modulus is dominated by segmental relaxations. A semiquantitative approach invoking relaxation times τ_BLS_ = (2*π f*_BLS_)^−1^ ≈ τ_segmental_ = *De*/*ε̇*, where *De* is the Deborah number leads to *ε̇* = 2π/*De f*_BLS_. Assuming *De* ∼ 10^4^ at such high BLS frequencies/strain rates, *ε̇* ∼ 10^–3^ *f*_BLS_.

Figure 3 compares the *n* = 8 data with BLS data without any shifting. The agreement is striking. To our knowledge, such comparisons are rare in the literature, as most experimental systems are compared with rheological experimental data many orders of magnitude apart. Even a frequency shift by 10^–3^ (as above calculation commands), amounts to numerical discrepancy of 1.5× between *C*_11_ computed versus measured, which still preserves the order of magnitude agreement.

**Figure 3:**
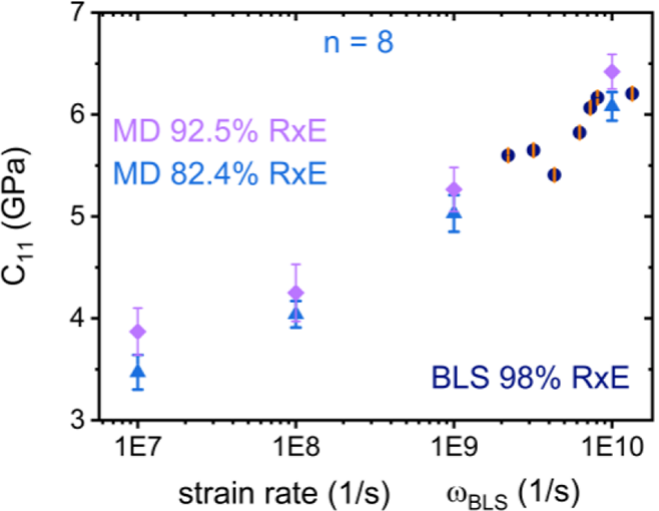
Comparison of the stiffness tensor element ***C***_**11**_ from MD simulations for *n* = 8 (blue triangles and purple diamonds) and Brillouin light scattering experiments (dark blue circles) for the experimentally matching system PEG-X500. Experimental error bars in orange are the size of the symbols.

Additional comparisons between simulated and experimental networks at the level of the glass transition temperature *T*_g_ also revealed agreement. Experimental PEG-X500 and PEG-X1k *T*_g_ values (Figure 4C–D, red x and orange square, respectively) were determined by DSC.^16^ Computationally, the *T*_g_ of the four networks was determined from the density change as the system was cooled down.^1,31^ The system’s temperature was elevated at 500 K and cooled with a step of 25K at a rate of either 0.5 or 0.05 K/ps as part of a sensitivity check, followed by a survey of dwell (equilibration) times ranging from 20 to 500 ps under NPT conditions at P = 1 atm. *T*_g_ was calculated for each configuration as the breaking point of a bilinear fit. Density was measured with 1 fs time step, and the average density was computed for each temperature. Survey results (Figure S3) establish convergence for the slower rate coupled with an equilibration time of >200 ps. For RxE ∼ 82%, results (Figure 4A–C) are reported for 500 ps dwell, whereas for RxE 92.5% (Figure 4D) for 200 ps. Density values are directly comparable (Figure 4C–D) to the experimental PEG-X500/X1k densities, as measured by weighing and image analysis of samples of known thickness. It is worth noting that experimental density values suffer from the mixing of small masses and spatial heterogeneities due to mixing. *T*_g_ values have too large error bars to be able to conclude a consistent decrease with increasing molar mass due to decreased cross-link density. For identical molar mass *n* = 8, the higher 92.5% RxE, more cross-linked sample exhibits a higher *T*_*g*_^92.5^ = 6 ± 11 °C > – 10.4 ± 2 °C = *T*_*g*_^82.4^ as expected. As MD *T*_g_ values are computed at rate of 0.05 K/ps = 3·10^12^ K/min which is 3·10^11^ K/min faster than the experimental 10 K/min of DSC, a well-documented shift of ∼3K/decade of cooling rate, predicted by the William–Landel–Ferry equation,^31^ i.e. a ∼33.9 K shift needs to be applied to allow the computational-experimental comparison. The adjusted MD *T*_*g*, adj_^92.5^ = −28 ± 11 °C is in very close proximity to the experimental PEG-X500 −40.6 °C value, validating the model and placing it on par with other literature-reported models. A second method for *T*_g_ calculation^32^ produced identical results (Figure S4).

**Figure 4:**
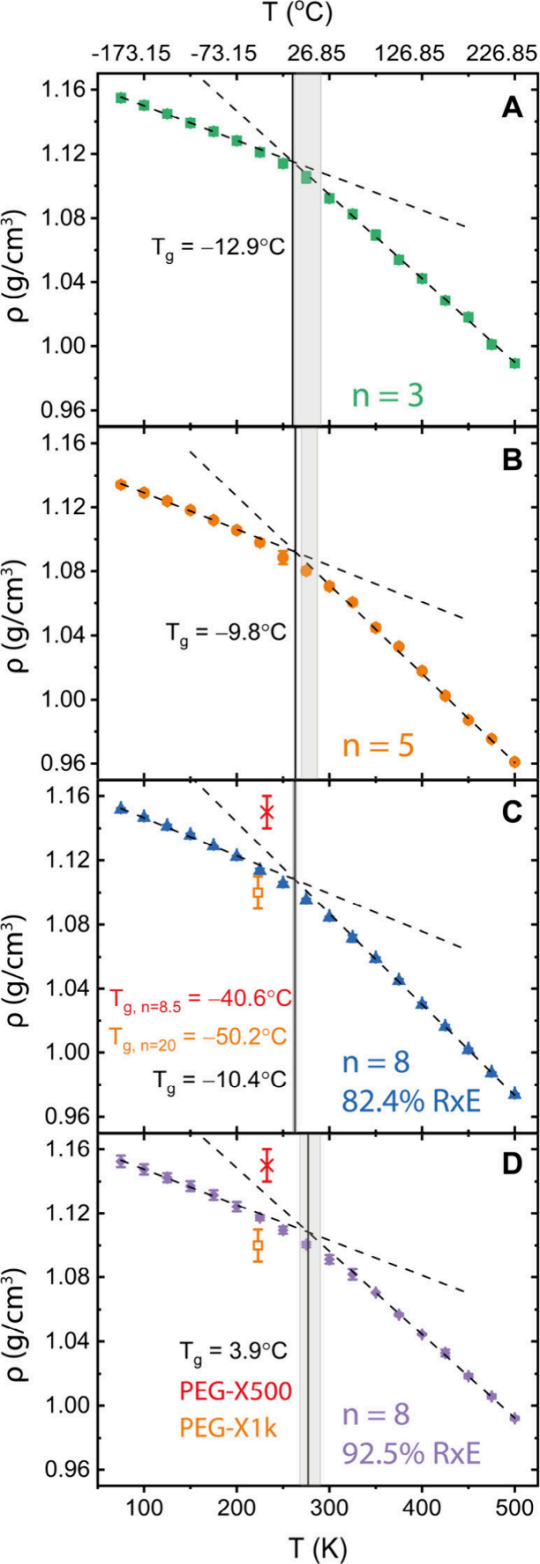
(A–D) Density versus temperature plots for (A) *n* = 3, (B) *n* = 5 and (C) *n* = 8 with approximately 82% DoX and (D) *n* = 8 with 92.5% RxE. Solid vertical lines mark the *T*_g_ found from MD simulations via the bilinear fit method on the averaged *ρ­(T)*. Shaded regions mark the mean ± st.dev. resulting from the *T*_g_ values of the three configurations. In (C–D), experimental (*T*_g_, ρ) values from the PEG-X500 (red x) and the PEG-X1k (orange square) are marked. Error bars are the size of the symbols.

A related quantity which links the bulk thermal property of *T*_g_ to the microscopic chain dynamics is the all-atom mean squared displacement (MSD).^33^ Simulations were conducted at the NPT ensemble, at 300 K and 1 atm for 100 ns with a time step of 1 fs. Atom trajectories were saved every 100 ps, and MSD was computed with the MDAnalysis Python tool (Figure 5).^34^ Systems of similar reaction extend have a similar fraction of atoms tethered to cross-link sites of low diffusivity. Thus, variations in MSD arise from the fluctuations of the atoms located in between cross-links or in non-cross-linked clusters. The latter account for only 3–7% of atoms (Table S1), the majority being single PEGDEs, single DABs or PEGDE-DAB dimers. For *n* = 8, extracted diffusion coefficient for the linear regime Δτ = [87, 96] ns is *D* ^*n*=8^ = 32.5 ± 0.5 Å^2^/*μs*. For *n* = 5 and 3, however, the Fickian regime is never reached, indicating either the need for an even longer simulation, or—as the scaling persists for the last 50 ns—an inherent inability of the cross-linked system to reach diffusive dynamics, attributed to: the higher fraction of low diffusivity atoms at/near cross-links as the molar mass of PEGDE decreases, our computational method which samples all atoms, and the inherent lack of free diffusion due to network connectivity.

**Figure 5:**
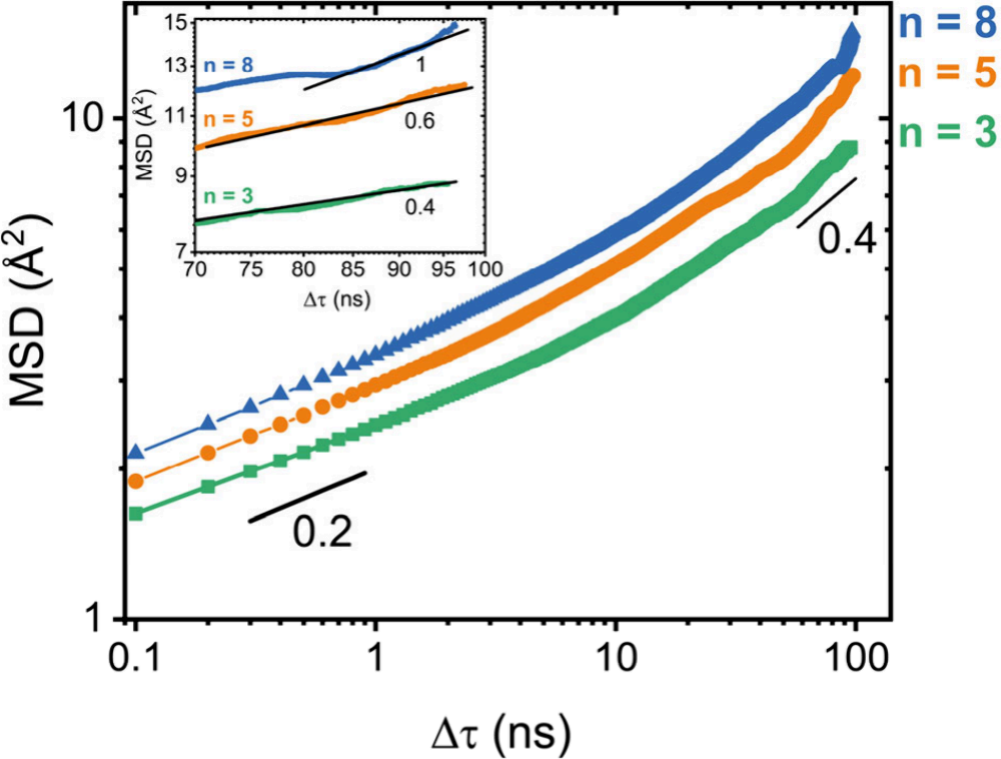
Mean squared displacement, log­(MSD), vs lag time, log­(Δτ), of all atoms with straight line fits for exponent extraction. Inset: log–log zoomed in view with individual exponents.

To our knowledge, this is the first time an MD simulation of epoxy networks with realistic molar masses is performed where the high frequency MD derived data are directly compared with experimental data from BLS and, on a positive note, are showing excellent agreement. Despite the differing methodology of obtaining the *C*_11_ (BLS versus tension), the key physical origin of both is segmental dynamics and thus can be comparable. Our comparison of simulations and BLS at high frequencies, paired with the discrepancy of mechanics at high and low frequencies, points toward bridging MD atomistic simulations and macroscopic experiments in the high frequency regime. We believe such a comparison is timely, in the context of the renewed interest in Brillouin spectroscopy applied to soft systems,^11–13^ hydrated and not. This work continues efforts to span frequencies in order to bridge low frequency with high frequency mechanical responses,^14,35^ comprehend regimes of appropriate comparisons between techniques, and, nowadays, take advantage of available high-performance computer resources. It opens the question of how hydrated systems would compare MD and BLS results, while it adds to the discussion of the less-studied nonglassy elastomers, which from a technical standpoint require longer equilibration times. It also validates the PCFF-IFF force field and provides benchmarks to convert from fully atomistic simulations of experimentally relevant oligo- and polymers to coarse-grained systems. Lastly, the particular epoxy network simulations are the necessary prerequisite for the construction of networks with complex connectivities, such as two types of bonding interactions, covalent epoxy-amine and sacrificial or metal coordinate bonds.^17,36^ We believe that a future combination of MD with experiments will shed light on the molecular mechanisms responsible for the increased toughness.

## Supplementary Material



## Notes

Network molecular composition, stress–strain curves, moduli values, *T*_g_ dependence on cooling rate and equilibration time, second method of *T*_g_ computation (PDF)
