# Supplementary material for: Elasticity and Dynamics of Elastomeric Epoxy Networks: Comparing Simulations and Experiments at High Frequency
Source: ACS Macro Lett. 2025 Nov 8;14(11):1783–8. doi: 10.1021/acsmacrolett.5c00517 (PMC12632164; doi:10.1021/acsmacrolett.5c00517)
Supplement: Supplementary file 1 [file mz5c00517_si_001.pdf]

Supplementary Information

Elasticity and dynamics of elastomeric epoxy networks: comparing simulations and experiments at high frequency

Iakovos Delasoudas[1], Spyros V. Kallivokas[2*], Emmanouela Filippidi[3,4*]

1. Dept. of Mechanical Engineering and Aeronautics, University of Patras, Patras, 26500, Greece
2. Computation-Based Science and Technology Research Center, The Cyprus Institute, Nicosia, 2121, Cyprus
3. Dept. of Materials Science and Engineering, University of Crete, Heraklion, 70013, Greece
4. Institute of Electronic Structure and Laser, FORTH, Heraklion, 70013, Greece

*Spyros V. Kallivokas s.kallivokas@cyi.ac.cy
*Filippidi Emmanouela filippidi@uoc.gr

Contents

I. Molecular composition of examined networks. Table S1.

II. Representative stress-strain curves of uniaxial molecular dynamics (MD) runs, Young's and Shear moduli Fig. S1, Tables S2 and S3.

III. Young's moduli of all systems as a function of uniaxial tensile strain rate. Fig. S2.

IV. MD Survey: Tg dependence on cooling rate and equilibration (dwell) time. Fig. S3.

V. Alternative method of Tg calculation based on discrete steps with identical results. Fig. S4.

I. Molecular composition of examined networks

Table S1. Number and type of molecules participating in a representative network with the total number of molecules being 128 PEGDE and 64 DAB for all simulations. RxE indicates the reaction extent, with each amine having two available reactions.

PEGDE n / RxE	unreacted PEGDE	unreacted DAB	PEGDE-DAB	PEGDE-DAB-PEGDE	(DAB-PEGDE)$_3$-DAB	in percolating network
3 / 82.4%	8	2	2	0	0	118 PEGDE 60 DAB
5 / 82.7%	0	2	4	0	0	124 PEGDE 58 DAB
8 / 82.4%	0	1	4	1	1	119 PEGDE 54 DAB
8 / 92.5%	0	0	0	0	0	all

II. Representative stress-strain curves of uniaxial molecular dynamics (MD) runs

We performed uniaxial tensile deformations in all three directions (xx, yy, zz) for each of the four tested strain rates at three statistically independent configurations for each of the networks, n = 3, 5, 8. We herein present representative curves from one configuration at all the strain rates. Faster strain rates result in higher Young's moduli, a typical response in cross-linked networks, due to the lack of time for relaxation. In (D), the slight increase of the stress compared to (C) is visible from the raw data, despite the noise in the data.

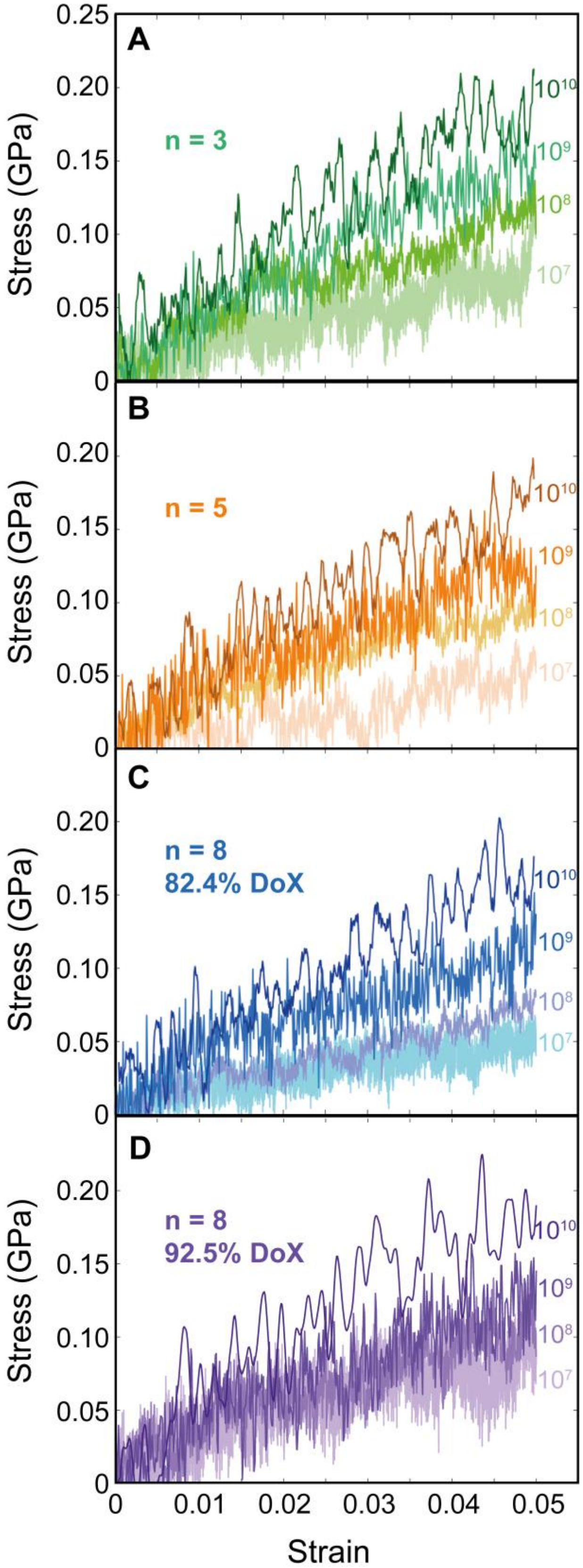

Figure S1. Representative curves drawn from one of the three realizations for each strain rate (10^7, 10^8, 10^9 and 10^{10} 1/s) shown next to the curve, for (A) n = 3 (green), (B) n = 5 (orange) and (C-D) n = 8 with different degrees of reaction extents: blue 82.4% and purple 92.5%.

Table S2. Averaged Young's moduli and standard deviation from uniaxial tensile MD simulations.

Strain rate / PEG-DE, n	10^7 s^{-1}	10^8 s^{-1}	10^9 s^{-1}	10^{10} s^{-1}
n = 3	1.5 ± 0.1 GPa	2.5 ± 0.2 GPa	3.3 ± 0.2 GPa	4.17 ± 0.15 GPa
n = 5	1.21 ± 0.15 GPa	2.1 ± 0.1 GPa	2.7 ± 0.2 GPa	3.8 ± 0.2 GPa
n = 8, 82.4% RxE	1.0 ± 0.1 GPa	1.6 ± 0.1 GPa	2.4 ± 0.2 GPa	3.5 ± 0.1 GPa
n = 8, 92.5% RxE	1.9 ± 0.3 GPa	2.2 ± 0.2 GPa	3.1 ± 0.2 GPa	3.8 ± 0.3 GPa

Table S3. Averaged shear moduli and standard deviation from MD simulations.

Strain rate / PEG-DE, n	10^7 s^{-1}	10^8 s^{-1}	10^9 s^{-1}	10^{10} s^{-1}
n = 3	0.4 ± 0.1 GPa	0.8 ± 0.1 GPa	1.27 ± 0.15 GPa	1.5 ± 0.1 GPa
n = 5	0.4 ± 0.1 GPa	0.7 ± 0.1 GPa	1.0 ± 0.1 GPa	1.4 ± 0.1 GPa
n = 8, 82.4% RxE	0.36 ± 0.05 GPa	0.55 ± 0.07 GPa	0.91 ± 0.08 GPa	1.3 ± 0.06 GPa

III. Young's moduli of all systems as a function of uniaxial tensile strain rate

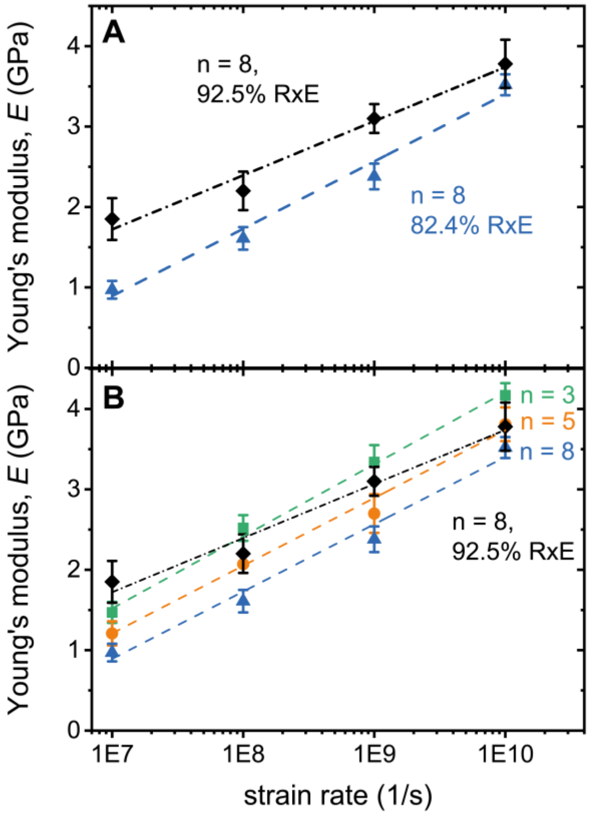

Figure S2. (A) Comparison of the Young's moduli between the n = 8, 92.5% RxE network (black dash-dot) and the n = 8, 82.4% RxE (blue dashed). (B) Comparison among all networks. The slope of the n = 8, 92.5% RxE data in the E vs log(strain rate) is 0.67 ± 0.08 GPa/decade of strain rate, whereas for the n = 8, 82.4% RxE is 0.84 ± 0.07 GPa/decade of strain rate.

The more cross-linked network for the same polymer length (n = 8, 92.5% versus the n = 8, 82.4%) forms a single percolating network (Table S1) and exhibits a higher Young's modulus (Fig. S2, top) as expected. There is a noticeable variation in the dependence on strain rate as demonstrated by the different slopes.

When the Young moduli of all studied networks are superimposed (Fig. S2, bottom) it appears that the mechanics of the more cross-linked network are intermediate to those of the stiffer networks n = 3 and n = 5, which are stiffer due to a higher cross-link density, due to the presence of shorter oligomer chain precursors.

IV. MD Survey: Tg dependence on cooling rate and dwell time

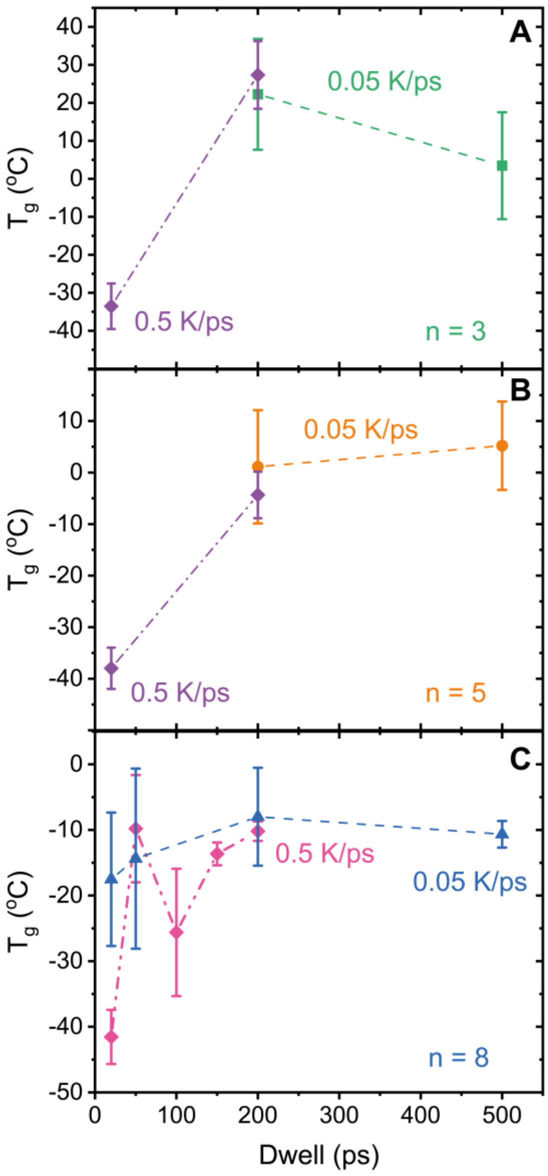

Computationally, the T_g of the three networks was determined from the density change as the system was cooled down.[1,2] The system's temperature was elevated at 500 K and cooled with a step of 25K at a rate of 0.5 K/ps or 10-fold slower at a rate of 0.05 K/ps, followed by various equilibration times under NPT conditions at P = 1 atm, ranging from 20 to 500 ps. Slower rates, e.g. by another decade, were unfortunately computationally too expensive to be systematically pursued.

Herein we present the comparative results of the variation of cooling rate and equilibration time on T_g for all three systems, with the bulk of the survey taking place at the n = 8 system, duee to our interest in comparing it with experiments. It is concluded that the slower rate and longer equilibration time (200 ps, 500 ps) contribute to improved convergence, i.e. smaller deviations in T_g values and similar values independent of the equilibration.

Figure S3. MD survey of cooling rates (0.5 K/ps, dash-dot line versus 0.05 K/ps, dashed line) and equilibration (dwell) times for the ~82% RxE (A) n = 3, (B) n = 5, (C) n = 8 systems.

V. Alternative method of Tg calculation based on discrete steps with identical results.

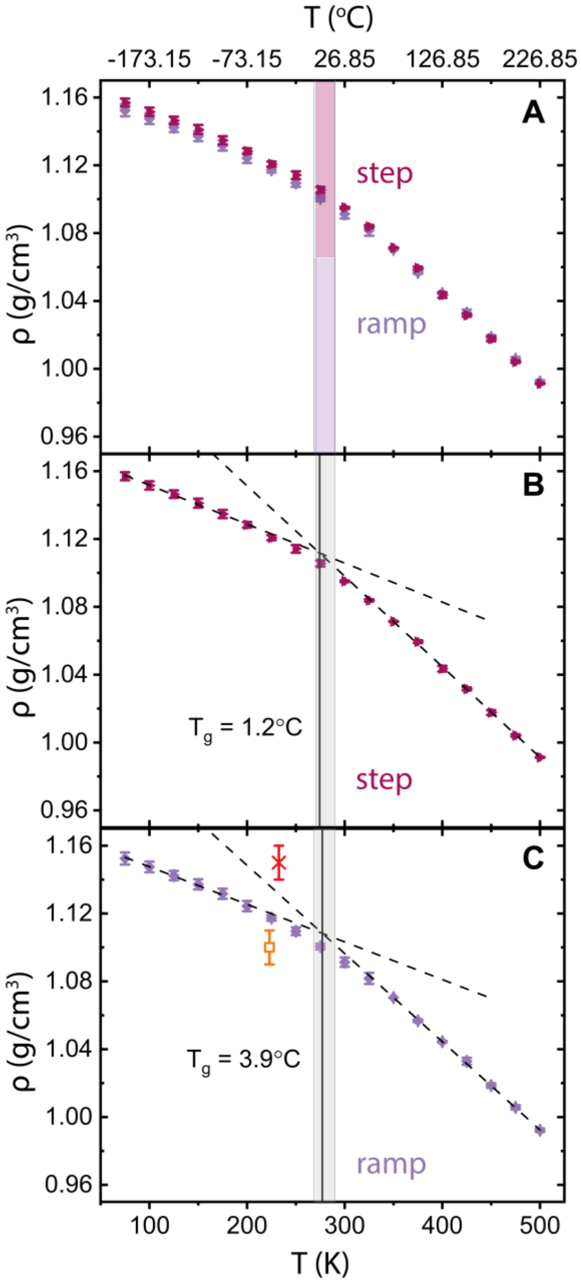

For the n = 8, 92.5% RxE system, we also applied another common methodology for calculating Tg.[3] This approach is still based on bilinear fitting of the density–temperature curve, but here the cooling rate is explicitly introduced through defined cooling steps, as opposed to a ramp used in the main text.

Specifically, the system was first heated to 500 K and then cooled in steps of 25 K until 75K. At each temperature, the system was equilibrated for 1 ns in the NPT ensemble. For every temperature step, the previously equilibrated configuration was used as the starting point for the next step. To calculate the average density at each temperature, we considered only the second half of the density trajectory. The results showed almost identical behavior with the methodology presented in the main manuscript.

Figure S4. Comparison of the ramp method (purple) and the step method (burgundy) for the MD calculation of the glass transition temperature (T_g) of the n = 8, 92.5% RxE. (A) Overlay of the (T, ρ_{mean}) points for the three configurations. Shaded regions correspond to the [T_g^{min}, T_g^{max}] from the T_g^{mean} ± standard deviation of the three configurations. Step (B) versus ramp method (C) with bilinear fits (dashed lines), intersection point (vertical solid line) and shaded areas as above.

References

(1) Meng, Z.; Bessa, M. A.; Xia, W.; Liu, W. K.; Keten, S. Predicting the Macroscopic Fracture Energy of Epoxy Resins from Atomistic Molecular Simulations. *Macromolecules* **2016**, *49* (24), 9474–9483. https://doi.org/10.1021/acs.macromol.6b01508.

(2) Li, C.; Strachan, A. Molecular Dynamics Predictions of Thermal and Mechanical Properties of Thermoset Polymer EPON862 / DETDA. *Polymer (Guildf).* **2011**, *52* (13), 2920–2928. https://doi.org/10.1016/j.polymer.2011.04.041.

(3) Siachouli, P.; Karadima, K. S.; Mavrantzas, V. G.; Pandis, S. N. The Effect of Functional Groups on the Glass Transition Temperature of Atmospheric Organic Compounds: A Molecular Dynamics Study. *Soft Matter* **2024**, *20* (24), 4783–4794. https://doi.org/10.1039/d4sm00405a.
